# Panacea, a semantic-enabled drug recommendations discovery framework

**DOI:** 10.1186/2041-1480-5-13

**Published:** 2014-03-06

**Authors:** Charalampos Doulaverakis, George Nikolaidis, Athanasios Kleontas, Ioannis Kompatsiaris

**Affiliations:** 1 Centre for Research and Technology Hellas, Information Technologies Institute, Thessaloniki, Greece; 2 Ergobyte S.A, Thessaloniki, Greece; 3 AHEPA University General Hospital of Thessaloniki, Thessaloniki, Greece

**Keywords:** Ontologies, Decision support, Rule-based reasoning, Drug recommendations

## Abstract

**Background:**

Personalized drug prescription can be benefited from the use of intelligent information management and sharing. International standard classifications and terminologies have been developed in order to provide unique and unambiguous information representation. Such standards can be used as the basis of automated decision support systems for providing drug-drug and drug-disease interaction discovery. Additionally, Semantic Web technologies have been proposed in earlier works, in order to support such systems.

**Results:**

The paper presents Panacea, a semantic framework capable of offering drug-drug and drug-diseases interaction discovery. For enabling this kind of service, medical information and terminology had to be translated to ontological terms and be appropriately coupled with medical knowledge of the field. International standard classifications and terminologies, provide the backbone of the common representation of medical data while the medical knowledge of drug interactions is represented by a rule base which makes use of the aforementioned standards. Representation is based on a lightweight ontology. A layered reasoning approach is implemented where at the first layer ontological inference is used in order to discover underlying knowledge, while at the second layer a two-step rule selection strategy is followed resulting in a computationally efficient reasoning approach. Details of the system architecture are presented while also giving an outline of the difficulties that had to be overcome.

**Conclusions:**

Panacea is evaluated both in terms of quality of recommendations against real clinical data and performance. The quality recommendation gave useful insights regarding requirements for real world deployment and revealed several parameters that affected the recommendation results. Performance-wise, Panacea is compared to a previous published work by the authors, a service for drug recommendations named GalenOWL, and presents their differences in modeling and approach to the problem, while also pinpointing the advantages of Panacea. Overall, the paper presents a framework for providing an efficient drug recommendations service where Semantic Web technologies are coupled with traditional business rule engines.

## Background

One of the health sectors where intelligent information management and information sharing compose valuable preconditions for the delivery of top quality services is personalized drug prescription. This is more evident in cases where more than one drug is required to be prescribed, a situation which is not uncommon, as drug interactions may appear. The problem is magnified by the wide range of available drug substances in combination with the various excipients in which the former are present.

If one takes into account that there exist more than 18,000 pharmaceutical substances, including their excipients, then it is clear that the continuous update of health care professionals is remarkably hard. Over this, the extensive literature makes discovery of relevant information a time consuming and difficult process, while the different terminologies that appear between sources add more burden on the efforts of medical professionals to study available information.

Semantic Web technologies can play an important role in the structural organization of the available medical information in a manner which will enable efficient discovery and access. Research projects funded for enabling Semantic Web technologies in the diagnosis and therapeutic procedures exist such as REMINE [1], PSIP [2], NeOn [3] and Active Semantic Documents [4] or works such as [5], but they don’t fully address the problem of automated drug prescription using drug-drug and drug-disease interactions.

Rule-based approaches have been proposed for addressing issues relating to biomedical ontologies research. It is common for ontologies written in expressive Semantic Web languages such as OWL^a^, to not be able to handle all requirements for capturing the knowledge in several biomedical and medicine domains. As a method for enriching the expressiveness of ontology languages, researchers have proposed the use of rules which act upon the defined ontological knowledge. According to [6], rules are helpful in the following situations relating to biomedical ontologies: defining “standard rules” for chaining ontology properties, “bridging rules” for reasoning across different domains, “mapping rules” for defining mappings between ontologies entities and “querying rules” for expressing complex queries upon ontologies. The author gives a thorough review of RuleML^b^ and SWRL^c^, the two major ontology rule languages, the available rule formation tools and the reasoners. Golbreich et al. [7] makes use of the outcomes of the previous paper to showcase the need for rules in biomedical applications with a use case of a brain anatomy definition, where a brain structure ontology is defined in OWL but rules describing the relationships between the properties and entities that are needed for correct annotation of MRI images. Another work citing the need for semantically enriched rules, where an ontology is coupled with SWRL rules for annotating pseudogenes and answering research questions, has been proposed in [8]. All the above papers present the need for extending ontologies with rules in order capture the knowledge of complex biomedical domains.

The paper presents Panacea, a semantic-enabled system for discovering drug recommendations and interactions. Panacea is based on experiences and lessons drawn from the development of GalenOWL [9], a similar system which had Semantic Web technologies in its core. As such, Panacea can be considered the evolution of GalenOWL in terms of design and scalability. Panacea makes use of established and standardized medical terminologies together with a rich knowledge base of drug-drug and drug-diseases interactions expressed as rules. Panacea is implemented having in mind scalability, completeness of results and responsiveness in query answering.

### Standard terminologies and semantic web

Standard terminologies and classifications in the medical domain have been developed in order to support information sharing and exchange and to enable a common expression of key concepts. Such is the case for example for the ICD-10^d^ (International Classification of Diseases) index of the World Health Organization (WHO) where “it is used to classify diseases and other health problems recorded on many types of health and vital records” across many countries. The classification is also used for storing and retrieval of diagnostic information and for the compilation of national statistics reports by the WHO members.

On the other hand, ontologies and the Semantic Web enable a common representation and understanding of knowledge. Ontologies can effectively capture a domain’s knowledge by “specifying the definitions of terms by describing their relationships with other terms”. A reasoner can be employed upon an ontology in order to uncover implicitly defined information while the expressiveness of ontologies can be further enriched by formulating rules in standard rule languages, such as RuleML or SWRL that are mentioned above, thus not sacrificing interoperability.

Panacea aims to combine and make use of the benefits of standard terminologies and Semantic Web technologies by enabling inference and rule-based reasoning on ontologies that have been expressed using the medical standards.

## Methods

### Architecture and functional design

The purpose of Panacea is to provide drug prescription recommendations based on a patient’s medical record, i.e. advise physicians to prescribe medications according to the drugs active substance indications and contraindications. For details regarding the initiative that triggered development of Panacea and the initial medical and pharmaceutical data that were available, the reader is encouraged to read [9].

**Figure 1:**
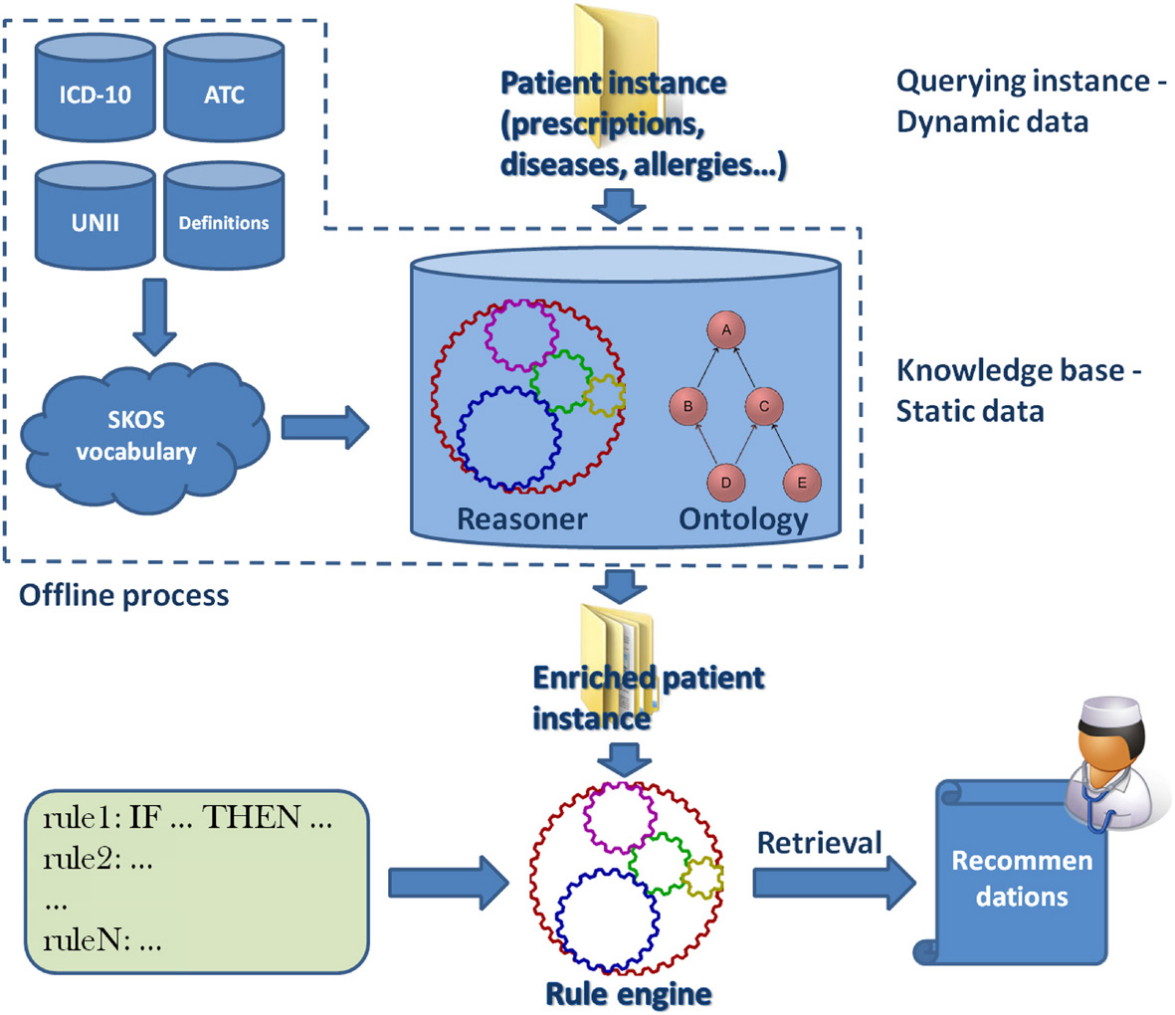
Panacea framework architecture and data flow.

Panacea follows a layered reasoning process which is depicted in Figure 1. During the start-up of the system, the medical terminologies, namely ATC^e^ (Anatomical Therapeutic Chemical), UNII^f^ (Unique Ingredient Identifier), ICD-10, ICTV^g^ (International Virus Taxonomy) and custom encodings, are transformed to semantic entities, using an appropriate vocabulary, and the initial ontology is constructed. The ontology binds to a reasoner to infer relations such as inheritance and unions. This process is performed once offline during initialization and the knowledge base is available to the system for further utilization. In order to get recommendations in Panacea, a patient instance with the appropriate medical record data is created and fed to the knowledge base. The reasoning process enriches the patient instance with inferred knowledge, thus making it explicit. On this enriched instance, and by utilizing a different reasoning process, the set of medical rules is applied upon. The result of this final stage of rule-based reasoning is the recommendations list which can be retrieved through SPARQL^h^ querying.

A key characteristic of the suggested architecture is that, regarding second level reasoning, the framework can utilize any rule-based reasoner or rule engine. Since all the inferred knowledge of the medical definitions and patient data is materialized in the knowledge base, the medical rules can be expressed and loaded in an appropriate rule engine. The rule engine could be an ontology reasoner or a business rule manager with appropriate customizations in the data structures. This approach helps in bringing together the best of both worlds: semantic and meaningful representation of data using Semantic Web technologies and the maturity of traditional rule engines in efficiently handling complex and large amounts of rules.

### Use case scenario

In order to demonstrate the benefits of the proposed semantic recommendation system, a use case regarding a possible scenario is described below. The codings in the parentheses represent the corresponding ICD-10 and ATC codes of diseases and drugs, respectively.

An elder man visits his family doctor complaining for pain in his right lower back and abdominal region which is accompanied with fever. After appropriate clinical examination, he is diagnosed with right pyelonephritis (ICD-10: N11.0). According to the patient’s medical history, he is suffering from chronic atrial fibrillation (ICD-10: I48.2) for which he receives clopidogrel (ATC: B01AC04), vertigo (ICD-10: H81.49) for which he receives cinnarizine (ATC: N07CA02), high arterial blood pressure (ICD-10: I10) for which he receives candesartan (ATC: C09CA06) and amlodipine (ATC: C08CA01), and diabetes mellitus (ICD-10: E11.9) for which he receives metformin (ATC: A10BA02) and sitagliptin (ATC: A10BH01). For the new condition of pyelonephritis that was diagnosed, the treating doctor must decide a number of things. Regarding the prescription for treating this new disease, the doctor has to decide which active substances to prescribe in order to treat the resulting inflammation, the cause of the inflammation, the back and abdominal pain and the resulting fever.

However, before a decision is made the following factors regarding the patient’s medical history should also be considered: 

•There should be a check for drug-drug interaction that the patient is taking, before the onset of the new condition (the pyelonephritis).

•There should be a check for drug-disease interaction of the drugs that the patient is already prescribed with the new condition.

•The new prescription has to be verified that it will not have adverse effects or interactions with the previously prescribed medication and with the patient’s medical history.

It is clear that the task for the doctor can be hard and a misjudgment could lead to wrong prescriptions. Using an automated drug recommendation system can minimize this risk. The recommendation system will use the input data and the pharmaceutical rules in order to propose a treatment that will be safe for the patient.

### Semantic transformations

Panacea is built on top of international standards of medical terminology in order to represent medical and pharmaceutical information. The following standard terminologies are used: 

**ICD-10**: International Classification of Diseases. It is used in Panacea for unique identification of diseases thus uniquely identifying drug indications and contraindications related to diseases. The latest 2010 version was used in this work.

**UNII**: Unique Ingredient Identifier. Used for the identification of active ingredients found in drugs. In Panacea it is used for uniquely identifying drug indications and contraindications related to ingredients. The 2013 index was used.

**ATC**: The Anatomical Therapeutic Chemical Classification is used for the classification of drugs. In Panacea it is used in similar fashion to UNII. The latest 2013 index was used.

**ICTV**: The International Committee on Taxonomy of Viruses indexing is used for the classification of viruses. In Panacea it is used in order to uniquely drug indications and contraindications related to viruses. The latest 2012 release was used.

Besides these international standards, a number of domain classifications have been declared and used in order to enhance the usability of the system or to represent data that are not included in the standards. These classifications act as supplementary to the standards.

*Substance*: As the use of encodings for drug ingredients is not convenient for humans, the identification of active substances is done using its common name references in medical bibliography. These names come from international standards such as the INN (International Nonproprietary Names) and others such as USAN (United States Adopted Name) or BAN (British Approved Name). Members of this identification list are substances such as *acetazolamide* or *isradipine*. In addition, substances correspond to ATC codes such that for example *acetazolamide* ≡ S01EC01. The substances are the actual recommendations of Panacea.

*Custom Concepts*: While the ATC, ICD-10, UNII and ICTV standards are complete, they are designed for use in contexts different from Panacea and drug recommendations, e.g. for annotation, search or information retrieval. As such, it is often desirable to enrich the knowledge base with information that, while not standard, will aid in the usability and overall efficiency of the system. Especially for medical/pharmaceutical rules formulation, it was found out that there were occasions that the definition of diseases, drugs or other was either absent, incomplete or too general to be useful for a rule definition. An example for the lack of a definition in ICD-10 is the absence of a precise and specific code for “Chronic obstructive pulmonary disease” or for “Hypertrophy (benign) of prostate” which is under the general code *N40 - Hyperplasia of prostate* among other hyperplasia conditions. For this reason, a number of custom concepts have been defined. Examples of such concepts is disease definition such as “Narcolepsy”, microorganisms such as “clostridium clostridiiformis” or medical acts such as “upper extremity arteriography”.

*Custom Concept Collections*: Certain “groups” of substances and/or diseases are frequently present in drug interactions and these groups are not recorded explicitly in any standardized classification, so it’s more convenient for medical use to specify these custom groups. These often used groups are termed “conditions” in Panacea and are defined by medical experts. A condition can appear as a premise in other condition definitions, as in the Custom Concept Collection *cardiac-rhythm-abnormalities*:

***cardiac-rhythm-abnormalities*** = cc:bradycardia | icd:R00 | cc:tachycardia | icd:O68.0 | icd:O68.2 where *cc:bradycardia* is defined as “icd:I49.5 | icd:R00.1 | icd:O68.0” and *cc:tachycardia* as “icd:R00.0 | icd:I49.5 | icd:I47 | icd:O68.0”. “icd:” stands for the ICD-10 namespace. 672 Custom Concept Collections have been defined and are used in this work.

#### SKOS vocabulary

In the approach followed in [9], the medical standards and the custom definitions were translated to OWL classes, primitive and defined. While this approach had the benefit of using the language’s semantics to model the available information, there were problems resulting from this design decision. One of the major issues was the difficulty in scaling the system. Until currently, very few reasoners are available that can efficiently handle the amount of class definitions and reasoning required to run the system, both in terms of memory consumption and speed.

In Panacea, a different approach was adopted. The SKOS^i^ (Simple Knowledge Organization System) vocabulary is a W3C (World Wide Web Consortium) recommendation, it’s built using RDFS (Resource Description Framework Schema) semantics and has been developed as a low-cost migration path for porting existing knowledge organization systems, such as thesauri, taxonomies, classification schemes and subject heading systems, to the Semantic Web. It enables a “lightweight” semantic representation of such knowledge systems and is a good match for the medical standards that are used in Panacea. As such, all the terminologies which are mentioned in the previous section have been transformed using the SKOS vocabulary automatically using a parser.

Comparing SKOS to the approach followed in [9], instead of representing the ATC, ICD-10 and UNII classifications as top-level classes, they are now represented as instances of the *skos:ConceptScheme* class. “*skos:*” stands for the SKOS namespace. Each entry in these classifications is represented as an instance of the *skos:Concept* class. The OWL class hierarchy of [9] is represented in Panacea using the properties *skos:broaderTransitive* and *skos:narrowerTransitive*, while the unions of classes for Custom Concepts Collections are represented using the *skos:member* property. Correspondence between the semantic transformation methodologies that were followed in the current work and in [9] is presented in Table 1.

**Table 1:** Correspondence between the semantic transformation in the early GalenOWL system and the proposed Panacea framework

	**GalenOWL**	**Panacea**
Annotation	rdfs:label	skos:prefLabel
Equivalence	owl:equivalentClass	skos:closeMatch
Custom collections	owl:unionOf	skos:member
Hierarchy	rdfs:subClassOf	skos:broaderTransitive

It is interesting to note that the SKOS vocabulary offers exactly what is needed in order to capture the semantics of the medical classifications without making sacrifices in expressiveness. One can argue that it can be considered more precise than the OWL expressions, as in the case of the similarity of Substances and ATC codes. This similarity is better represented by the *skos:closeMatch* relation than *owl:equivalentClass*. For Panacea a total of 64,658 definitions of classification codes have been expressed using SKOS.

### Panacea ontology and reasoning

**Figure 2:**
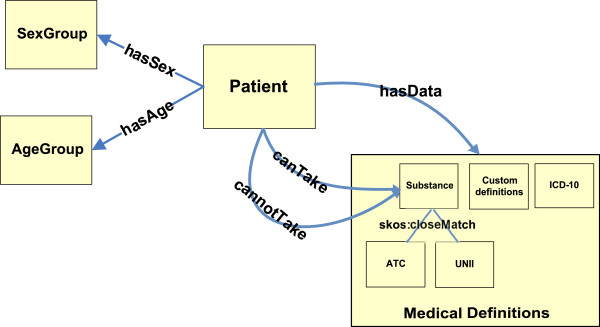
Panacea ontology.

The core ontology of Panacea is visualized in Figure 2. The aforementioned SKOS ontologies were imported to the Panacea core ontology under the *MedicalDefinitions* class. The Patient class holds the patient instances and is connected to the *MedicalDefinitions* class with the *hasData* properties. The patient recommendations, indications and contraindications, regarding substances that should and should not be prescribed are expressed with the *canTake* and *cannotTake* properties, respectively. The patients age group and sex group are expressed through the *hasAgeGroup* and *hasSexGroup* properties.

#### Medical reasoning

When querying the system for recommendations, a patient instance is created with the initial patient data (through the *hasData*, *hasAgeGroup* and *hasSexGroup* properties) and is loaded in the knowledge base. The reasoner, using RDFS inference and a small number of additional rules, infers all the implicit patient data. As an example consider a patient who suffers from a form of thrombocytopenia. An instance is created with the property *<pnc:patient pnc:hasData icd:D69.6>*. The reasoner through the skos:broaderTransitive relation will infer the triples *<pnc:patient pnc:hasData icd:D69>, <pnc:patient pnc:hasData icd:D65-D69>, <pnc:patient pnc:hasData icd:D50-D89 >*. Additionally, the custom collection definition of *pnc-cc:deficiency-bone-marrow* has *icd:D69.6* as one of its members so the triplet *<pnc:patient pnc:hasData pnc- cc:deficiency-bone-marrow>* will also be inferred. At the end, the patient instance will be enriched with all the underlying implicit information.

#### Rule-based reasoning

Drug recommendations in Panacea are generated using a rule-based approach. The rules express the indications and contraindications of drug substances while their premises are the medical definitions and the patients’ age and sex group. The rules use the logical operators *and* (&) and *or* (|) and parentheses. An example of a rule is for the substance “ lisuride” which is expressed as

***lisuride*** = icd:E22.0 | (icd:E22.1 & (icd:N91.0 | icd:N97)), ageGroup=*adult* or *elder*

The above rule reads that the substance “lisuride” is recommended for adult and elder patients who suffer from E22.0, OR suffer from E22.1 AND one of the N91.0 OR N97. For using these rules, they have to be properly parsed and transformed in order to match the knowledge base and the enriched, with implicit knowledge, patient instance. The proposed rule structure allows modifications to specific rules without the changes affecting the rest of the rule base. This enables the rule base to be up-to-date with the latest clinical advancements, which is a requirement as clinical pharmacology and medicine are constantly evolving. Analyzing Panacea’s architecture in Figure 1 it can be seen that due to the layered reasoning approach, the knowledge base (medical definitions + reasoner) is actually used for producing the enriched patient instance. This means that the instance can be fed to a rule reasoner which has appropriately loaded the medical-pharmaceutical rules, without the reasoner having to communicate with the knowledge base for further utilization. Using this approach and with proper modifications, any rule engine can be used to produce the drug recommendations. To demonstrate this ability, two separate rule engine integrations have been developed and are presented below. The medical rule base consists of 1,342 rules which were extracted and encoded directly from official documents, such as Summary of Product Characteristics (SPC) and Patient Information Leaflets (PIL), regarding drug indications, contraindications, interactions and dosage. The validity of the rule base has already been assessed in [9].

It should be noted that work is under way in order to add more functionalities in the drug proposed recommendation system. One of these is the ability to offer additional information such as the proposed dosage for a recommended substance. In order to accomplish such a task, the pharmaceutical rules are being enriched with clinical variables, other than sex and age group, that are important. These variables include somatometric characteristics such as height and body weight, creatinin clearance (useful for calculating the dosage for antineoplasmic drugs) and the disease itself as a substance could be indicated at a specific dosage to treat a certain disease, but a different dosage is recommended for another disease.

#### Jena rule engine

For using the rule engine of the Apache Jena API^j^ the rules had to be translated to the Jena rule language. An automated parser was developed for this purpose. As for most semantic rule reasoners, OR clauses are not allowed in a rule definition so separate rules had to be expressed for every premise that was OR’ed in the original rule base. For example, the rule for “lisuride” was expressed by 3 different rules: 

This rule expansion resulted in a total of 6,451 rules to be expressed in the Jena language. Trying to load the whole rule base and performing inference for recommendations proved inefficient for real time use, requiring on average as much as 8 seconds. In order to tackle this issue a coarse rule selection phase was introduced. The selection was executed in 2 iterations. During the first iteration, a subset  of candidate rules is created from the initial rule base, that match the patient’s sex and age group. This subset is selected for further processing. In the second iteration, rules from  that contain at least one of the patient’s data, i.e. a *skos* term, in their premises are singled out and a final set R⊆A is created from them. Remembering that the implicit knowledge extraction was performed during the introduction of the patient instance to the reasoning framework, creation of  is actually a simple and fast process. It merely requires string matching and all the whole processing is executed in memory. As a result the overall burden that is added to the whole reasoning process is minimal. From the initial rule base of 6,451 rules it is common for  to contain as less as 50 rules, whose evaluation is much more efficient. Rule execution is performed with the Jena rule engine and the patient instance is modified and now contains the drug recommendations. These recommendations are retrieved through SPARQL querying, using Jena’s query engine. The advantage of the Jena engine is that it can readily consume the patient instance for producing the recommendations.

#### Drools rule engine

As an alternative approach, the Drools^k^ business rule engine was used. In contrary to Jena, Drools could not directly use the patient instance for performing reasoning. For this purpose, the instance was transformed to a Java bean, where the properties of the ontology Patient class are mapped to Java methods using the JenaBean API^l^. A similar approach for integrating Jena and Drools was used in [10]. The Drools rule language permits the use of OR’ed clauses in the body, so the 1,342 original medical rules were translated to the same amount of rules in Drools, using an automated parser similar to the one used in the Jena approach. For example, the rule for “lisuride” from the previous paragraph was expressed as: 

Execution was straightforward with no preprocessing required. Drools is optimized for handling large rule bases, so no rule pre-selection step was required as this would have little impact in reasoning efficiency. The result of this reasoning process is a modified patient Java bean with the drug recommendations. The Java bean is transformed to Jena model instance and SPARQL querying for retrieving the recommendations is possible. What this approach demonstrates is that it’s possible to integrate business rule engines as reasoners in the framework, thus being able to make use of the high efficiency and optimizations of these engines with the semantic description and interpretation of data.

## Evaluation and discussion

The evaluation of Panacea was performed using two different approaches. One approach assesses the quality of drug recommendations while the other assesses the efficiency of the developed system in terms of computational requirements and performance which are of importance when a system is launched in a production environment. Both approaches are detailed in the next Sections.

### Quality evaluation

The first approach involved the evaluation regarding real clinical data from treated patients in a hospital environment. 21 anonymized patient medical record files (cases) from the AHEPA^m^ University Hospital of Thessaloniki were gathered and an analysis was performed on then. Data regarding the patients medical history (medication that the patients are taking and active diseases that they suffer from), diagnosis related to condition that lead the patients visiting the hospital and the medication that was actually prescribed, were gathered. These data formed the basis against which the prescription recommendations, that Panacea generates, were compared to. In addition, all patient data (existing diseases, current medication, newly diagnosed disease(s), new medication) were used by Panacea in order to discover possible interaction and/or contra-indications that were either missed or disregarded by the treating physicians. As such, the comparison identified the following: 

•Average number of identified drug-drug interactions per case

•Average number of identified drug-drug contraindications per case

•Average number of identified drug-disease interactions per case

•Average number of identified drug-disease contraindications per case

•Percentage of agreement between the automatically generated drug recommendations vs actual prescription for all cases, i.e. how many of the recommendations Panacea generated agreed with the prescribed drugs, for the newly diagnosed disease(s).

Although it doesn’t affect the final results, it should be noted that the evaluation took place using Drools as a rule engine. The identified interactions and contraindications per case are displayed in Table 2. For most of the cases where contraindicated drugs were prescribed, it was either done deliberately, e.g. a patient suffering from brain ischemia was administered *tinzaparin* and *acetylsalicylic acid* for their (normally contraindicated) enhanced blood thinning effect when combined, or because the contraindications were deemed unimportant related to the patient’s critical condition. However there were a few cases where the administration of contraindicated drugs couldn’t be justified. For these cases we can assume that the physicians didn’t have knowledge or made an error during prescription. The use of an automated recommendation system, such as the one presented, could have prevented such errors.

**Table 2:** Discovery of drug interactions and contraindications against real clinical data

	**Drug-drug**	**Drug-drug**	**Drug-disease**	**Drug-disease**
	**interactions/case**	**contraind./case**	**interactions/case**	**contraind./case**
	3.28	0.43	4.48	0.24
Actual prescribed	7.38			
drugs/case				

All numbers are average.

At the next stage of the evaluation, the patients’ medical history (active diseases and current medication) and the current diagnosis was taken into account in order to generate automatic recommendations from Panacea. These recommendations were compared to the prescription that the patients received from the hospital. Using this comparison, Panacea managed to match the 67.1% of the prescribed drugs (Table 3). While a level close to 100% would have been expected in a fully controlled environment where all prescriptions are given according to formal medical guidelines, this was not achieved due to several parameters that their role was revealed during the evaluation. On average, Panacea recommended 62 different drugs per case.

**Table 3:** Matching recommendations

**Total administered drugs**	**76**
Matching recommendations	51
Av. recommendations/case	62
Percentage (matching/administered)	67.1%

Several reasons that affected the recommendation results have been identified. One of them is that in its current form, Panacea rules expressing drug interactions are binary, thus an interaction either exists or not without having the means to express its level of effect (how important it is considered). The presence of an interaction, as unimportant as it may be, will exclude a relevant active substance from recommendation while in principle the benefit of administering the substance might be more important than a possible side effect. An example in the current evaluation dataset is the administration of the substance *Budesonide* to a patient whose current medication contains *Acetylsalicylic acid* and has been newly diagnosed with “Pneumonia”. The treating physician made the decision that the benefit from receiving the *Budesonide* was greater that the risk of possible interactions. Experienced physicians are in a position to effectively make this kind of judgments.

Secondly, there is the possibility that patient data might have been logged inaccurately, e.g. a patient was suffering from *Sepsis due to Staphylococcus aureus (ICD-10: A41.0)* while *Sepsis (ICD-10: A41)* was registered as diagnosis, or some information from the diagnosis is missing, e.g. a patient was diagnosed with “Pneumonia”, but the causing bacteria strand (staphylococcus) was omitted. Such inaccuracies or missing information affect the automated recommendations results as the recommendation rules have to match all premises in order to produce the results.

An additional cause for decreased accuracy has been mentioned earlier in the Section, i.e. contraindicated drugs could be prescribed because they would cause a desirable side effect that will be of benefit to the patient as is the case of simultaneous delivery of *tinzaparin* and *acetylsalicylic acid* in a patient with ischemic stroke. Especially for these situations, the experience of the treating physician plays an important role for such decisions. Panacea is the analogy of an inexperienced physician who goes “by the book” in contrast to an experienced physician who has the knowledge to make a successful compromise between possible risks (from interactions) and benefits (from the drug).

The above evaluation gave useful insights on ways where Panacea could be improved in order to be a valuable tool in a physician’s arsenal. Panacea has proven quite effective in identifying drug interactions and contraindications. Regarding the potential for actual drug recommendation, although Panacea managed to propose various possible treating drugs according to medical record data, these recommendations in some cases varied from the actual drug prescription that the patients received. Several factors that influenced the results have been identified. One major issue seems to be that in many cases the diagnosis is given in a general and unclear statement, e.g. respiratory infections or sepsis, while recommendation rules are concretely structured and give answers to specific disease diagnosis. For such recommendation systems to be efficient, diagnosis as general as e.g. “respiratory infection” are not adequate. The respiratory system starts from the nostrils and ends in the pulmonary alveoli and there are numerous ICD-10 codes that describe every individual infection in the respiratory system, as there are numerous bacteria or viruses that causes these infections. These combinations of diseases and causes are precisely encoded in Panacea’s rules and a precise diagnosis would generate the exact recommendation. However, a treating physician is often not in a position to exactly know the topology and the cause of an infection so it is common to prescribe drugs that cover most of the possible combinations. This generalization of something specific, e.g. pneumonia due to streptococcus, to something more general, e.g. respiratory infection, is what affects the recommendations results. Issues such as the above should be taken under consideration during the future development of Panacea.

### Performance evaluation

For evaluating the framework in terms of performance, a comparison was made between the two approaches for the final stage reasoning and with GalenOWL (with values taken from [9]). The comparisons were focused on the usability of the framework in a production environment as the rule base has been validated in [9]. Three parameters were measured. These were initialization time, the time to get the system up and running, memory consumption after initialization, and query response time, i.e. the time that is needed to have the rule base executed and the results retrieved. Results are shown in Table 4.

**Table 4:** Evaluation between the 2 Panacea reasoning approaches and GalenOWL

	**Panacea-Jena**	**Panacea-Drools**	**GalenOWL**
Initialization	32.0 s	34.7 s	148 s
time			
Memory	169 MB	280 MB	649 MB
consumption			
of which rule base	0 MB	111 MB	—
consumes			
Query response	47 ms	5 ms	16 ms
time			

There are some points to discuss in the table results. Initialization involves loading the ontology in memory, performing inference, and preparing the medical rule base for patient data reasoning. In the Jena implementation, the rule base is processed and loaded only after the patient instance has been introduced to the system, while the Drools implementation loads the whole rule base on the engine before any patient data are introduced. As a result, Drools appears slower than the Jena approach regarding initialization. For the same reason, memory consumption appears greater for Drools. This metric corresponds to memory consumption from initialization to recommendations retrieval. While in Drools the whole rule base is loaded on memory, in Jena the approach was to load a small subset of the rule base that could possibly match the patient data, which leads to a smaller memory footprint. Finally, for query response the advantage is with Drools, as was expected, mainly due to the fact that Drools is a dedicated rule engine while Jena’s focus is not at providing a state of the art reasoner and rule engine, but a versatile API for ontology management.

Numerically, the Jena approach seems to be more efficient than Drools, apart from the query execution time but for which the difference is not important. However, while for the present knowledge base Jena seems to perform better, this fact could change as more and more rules are added. It is estimated that eventually at its final stage, Panacea will incorporate more than 9,000 drug-drug and drug-disease interactions. As already said, Jena is more focused as an ontology API and less as an efficient rule engine which could eventually lead to scaling problems. On the other hand, scaling with Drools is not an issue. The value of business rule engines as Semantic Web reasoners has been previously exploited using approaches such as [11], where the authors implemented two OWL2-RL [12] reasoners using the Drools and Jess rule engines respectively. The use of traditional rule engines with the Semantic Web technologies brings together the best of both worlds, i.e. increased efficiency coupled with interoperability and semantic annotation of information.

What is also noticeable from Table 4 is the decreased memory requirement of Panacea compared to the previous OWL-based GalenOWL system, although the two approaches offer very similar functionality. As a result of this achievement, Panacea can accommodate a far greater knowledge base thus supporting the claim of increased scalability.

Panacea will eventually be offered as a service with potential customers being health care professionals. Other possible exploitation routes are being investigated such as integration to patient management systems in health clinics. The use of personalized drug prescription systems, as Panacea, in everyday practice will have advantages to the society and the economy. A major benefit from the use of such systems is the reduction of medical costs through rational drug prescriptions that personalized drug prescription allows [13]. Another benefit is a positive effect in public health with reduction of outbreaks relating to drug interactions or adverse effects [14]. All knowledge regarding drug information is encoded and is available to the experts in order to aid them during prescriptions thus acting as decision support systems. It should be stressed out that drug recommendation systems do not aim to replace medical experts but to support them in their practice.

A limitation of the proposed approach is that a rather large amount of manual effort by experts is required in order to populate and enrich the rule base. Although the semantic technologies that have been employed can make rule authoring simpler, no automated method for pharmaceutical rule generation has been integrated. However, one would argue that since rule authoring is performed by experts then the rules are verified and guaranteed to be correct. Even if an automated method, such as rule mining, had been implemented, the generated rules would still have to be verified be an expert in the field. Manual verification, although less intensive, would still be required.

## Conclusions

The paper presented Panacea, a framework for semantic-enabled drug recommendations discovery. The framework utilizes a layered reasoning approach were the medical ontology and the patient data instances are fed to an extended RDFS reasoner in order to infer implicit knowledge. Drug recommendations are generated using the second reasoning layer where any common rule engine can be used. As a proof of concept implementation, the Jena reasoner and the Drools rule engine has been integrated. Two different evaluations were conducted. One performance evaluation regarding requirements and efficiency of the proposed approach, and a quality evaluation regarding the system’s outcome in terms of real clinical data. The quality evaluation gave insights regarding possible extensions that could make the system more in line with current clinical practice. Future work on Panacea will focus on providing ways to address the issues uncovered during the quality evaluation and provide results that more closely match a physician’s decision. These could include improvements such as the weighting of interactions and contraindications according to a severity observation and probabilistic inference based on these weights. To this end, Drools is being extended with a fuzzy reasoning engine [15], which while it’s still in development, it’s actively supported and it is mature enough to be able to use it as a testing framework. Finally, the addition of dosage recommendations in the rules is an ongoing work.

## Endnotes

^a^ OWL - Web Ontology Language, http://www.w3.org/TR/owl2-overview/

^b^ RuleML - Rule Markup Language, http://www.ruleml.org

^c^ SWRL - Semantic Web Rule Language, http://www.w3.org/Submission/SWRL/

^d^ ICD-10 - International Classification of Diseases, http://www.who.int/classifications/icd/en/

^e^ ATC - Anatomical Therapeutic Chemical classification, http://www.whocc.no/atc/structure_and_principles/

^f^ UNII - Unique Ingredient Identifier, http://www.fda.gov/ForIndustry/DataStandards/ SubstanceRegistrationSystem-UniqueIngredientIdentifierUNII/default.htm

^g^ ICTV - International Virus Taxonomy, http://www.ictvonline.org/virusTaxonomy.asp

^h^ SPARQL - Query language for RDF, http://www.w3.org/TR/rdf-sparql-query/

^i^ SKOS - Simple Knowledge Organization System, http://www.w3.org/2009/08/skos-reference/skos.html

^j^ Apache Jena - Semantic Web framework, http://jena.apache.org/

^k^ Drools - Business logic integration platform, http://www.jboss.org/drools

^l^ JenaBean API, http://code.google.com/p/jenabean/

^m^ AHEPA University Hospital of Thessaloniki, http://www.ahepahosp.gr

## Abbreviations

ATC: Anatomical Therapeutic Chemical; BAN: British Approved Name; ICD-10: International Classification of Diseases; ICTV: International Committee on Taxonomy of Viruses; INN: International Nonproprietary Names; OWL: Web Ontology Language; PIL: Patient Information Leaflets; RDFS: Resource Description Framework Schema; RuleML: Rule Markup Language; SKOS: Simple Knowledge Organization System; SPC: Summary of Product Characteristics; SWRL: Semantic Web Rule Language; UNII: Unique Ingredient Identifier; USAN: United States Adopted Name; W3C: World Wide Web Consortium; WHO: World Health Organization.

## Competing interests

The authors declare that they have no competing interests.

## Authors’ contributions

CD was the lead developer of the Panacea framework and wrote major parts of the manuscript, GN contributed to the integration of the Drools rule engine and to the writing of the manuscript, AK contributed to the evaluation of the framework and to the writing of the manuscript and IK had the scientific lead and contributed to the writing of the manuscript. All authors read and approved the final manuscript.

## References

[B1] Ceusters W Capolupo M De Moor G Devlies J **Introducing realist ontology for the representation of adverse events** Proceedings of the 2008 Conference on Formal Ontology in Information Systems (FOIS 2008) 2008 The Netherlands: Amsterdam 237 250

[B2] Beuscart R McNair P Brender J PSIP consortium **Patient safety through intelligent procedures in medication: the PSIP project** Stud Health Technol Inform 2009 148 6 13 19745230

[B3] Suarez-Figueroa MC Gomez-Perez A **NeOn methodology for building ontology networks: a scenario-based methodology** Proceedings of the International Conference on Software, Services & Semantic Technologies 2008 Bulgaria: Sofia

[B4] Sheth A **Semantic web & semantic web services: Applications in healthcare and scientific research, keynote talk** IFIP Working Conference on Industrial Applications of Semantic Web 2005 Finland: Jyvaskyla

[B5] Adnan M Warren J Orr M **Ontology based semantic recommendations for discharge summary medication information for patients** Computer-Based Medical Systems (CBMS), 2010 IEEE 23rd International Symposium On 2010 456 461 doi:10.1109/CBMS.2010.6042688

[B6] Golbreich C Antoniou G Boley H **Combining rule and ontology reasoners for the semantic web, invited talk, rules and rule markup languages for the semantic web** LNCS 3323 2004 Hiroshima, Japan: Springer

[B7] Golbreich C Dameron O Bierlaire O Gibaud B **What reasoning support for ontology and rules? the brain anatomy case study** Workshop on OWL Experiences and Directions 2005 Ireland: Galway

[B8] Holford M Khurana E Cheung K-H Gerstein M **Using semantic web rules to reason on an ontology of pseudogenes** Bioinformatics [ISMB] 2010 26 12 71 78 10.1093/bioinformatics/btq173 PMC2881358 20529940

[B9] Doulaverakis C Nikolaidis G Kleontas A Kompatsiaris I **GalenOWL: Ontology based drug recommendations discovery** J Biomed Semantics 2012 3 14 doi: 10.1186/2041-1480-3-14 10.1186/2041-1480-3-14 PMC3561213 23256945

[B10] Bragaglia S Chesani F Ciampolini A Mello P Montali M Sottara D **An hybrid architecture integrating forward rules with fuzzy ontological reasoning** Proceedings of the 5th International Conference on Hybrid Artificial Intelligence Systems - Volume Part I. HAIS’10 2010 Berlin, Heidelberg: Springer 438 445 doi:10.1007/978-3-642-13769-3_53. http://dx.doi.org/10.1007/978-3-642-13769-3_53

[B11] O’Connor M Das A **A pair of OWL 2 RL reasoners** Proceedings of OWL: Experiences and Directions Workshop 2012 (OWLED-2012) 2012 Greece: Heraklion

[B12] Motik B Grau BC Horrocks I Wu Z Fokoue A Lutz C **OWL 2 Web Ontology Language Profiles, W3C recommendation** 2009 http://www.w3.org/TR/owl2-profiles/

[B13] Fischer M Vogeli C Stedman M Ferris T Brookhart M Weissman J **Effect of electronic prescribing with formulary decision support on medication use and cost** Archives Intern Med 2008 168 22 2433 2439 doi:10.1001/archinte.168.22.2433. /data/Journals/INTEMED/5729/ioi80125_2433_2439.pdf 10.1001/archinte.168.22.2433 19064827

[B14] Ammenwerth E Schnell-Inderst P Machan C Siebert U **The effect of electronic prescribing on medication errors and adverse drug events: A systematic review** J Am Med Inform Assoc 2008 15 5 585 600 doi:10.1197/jamia.M2667 10.1197/jamia.M2667 18579832 PMC2528040

[B15] Sottara D Mello P Proctor M **Adding uncertainty to a Rete-OO inference engine** Proc. of the International Symposium on Rule Representation, Interchange and Reasoning on the Web. RuleML ’08, 2008 Orlando, FL, USA 104 118

